# Development of an experimental apparatus to observe ultrafast phenomena by tender X-ray absorption spectroscopy at PAL-XFEL

**DOI:** 10.1107/S1600577521011449

**Published:** 2022-01-01

**Authors:** Yujin Kim, Daewoong Nam, Rory Ma, Sangsoo Kim, Myung-jin Kim, Jinhong Kim, Intae Eom, Jae Hyuk Lee, Tae Kyu Kim

**Affiliations:** a Department of Chemistry, Yonsei University, Seoul 03772, Republic of Korea; b Pohang Accelerator Laboratory, POSTECH, Pohang, Gyeongbuk 37673, Republic of Korea; c Photon Science Center, POSTECH, Pohang, Gyeongbuk 37673, Republic of Korea

**Keywords:** X-ray free-electron lasers, X-ray absorption spectroscopy, tender X-ray, time-resolved X-ray spectroscopy, PAL-XFEL

## Abstract

Understanding the ultrafast dynamics of molecules is of fundamental importance. Time-resolved X-ray absorption spectroscopy (TR-XAS) is a powerful spectroscopic technique for unveiling the time-dependent structural and electronic information of molecules that has been widely applied in various fields. Herein, the design and technical achievement of a newly developed experimental apparatus for TR-XAS measurements in the tender X-ray range with X-ray free-electron lasers (XFELs) at the Pohang Accelerator Laboratory XFEL (PAL-XFEL) are described. Femtosecond TR-XAS measurements were conducted at the Ru *L*_3_-edge of well known photosensitizer tris­(bi­pyridine)­ruthenium(II) chloride ([Ru(bpy)_3_]^2+^) in water. The results indicate ultrafast photoinduced electron transfer from the Ru center to the ligand, which demonstrates that the newly designed setup is applicable for monitoring ultrafast reactions in the femtosecond domain.

## Abstract 2

Tender X-ray absorption spectroscopy was successfully performed with the newly developed experimental apparatus at PAL-XFEL.

## 1. Introduction

The dynamics of chemical reactions involve the coupled motion of electrons and nuclei on femtosecond time scales (Lemke *et al.*, 2013 ▸), and fully understanding these complex dynamics has been a challenge for both experimental and theoretical studies. Femtosecond optical spectroscopy has been used to monitor changes in valence electronic states of molecules and the vibrational states of reaction intermediates or products (Cammarata *et al.*, 2014 ▸). However, optical probes only provide indirect information because visible light interacts with the extended electronic structure (Schoenlein *et al.*, 2000 ▸). Thus, experimental tools needed to study molecular and valence electronic structures along the strongly interacting potential energy surfaces are required.

X-ray absorption spectroscopy (XAS) probes core-level transitions for deducing element-specific information and observing tiny changes in the electronic structure of a sample in various states (from gas to solid) since the discontinuity of the absorption spectrum with incident photon energy in the X-ray region makes distinct differences observable through photon-out spectroscopy of samples (Kapilashrami *et al.*, 2014 ▸; De Groot, 2001 ▸; Bressler & Chergui, 2004 ▸). In time-resolved XAS (TR-XAS), the system is excited by using a laser pump pulse followed by an X-ray probe pulse after a time delay, which enables real-time monitoring of ultrafast photoinduced reactions (Chen, 2005 ▸; Chen *et al.*, 2014 ▸). While generating ultrashort X-ray pulses with X-ray free-electron lasers (XFELs), synchrotrons with time-slice methods, and high-harmonic generation including plasma X-ray sources, XAS combined with a femtosecond laser has been intensively applied to reveal ultrafast phenomena with unprecedented temporal resolution (Attar *et al.*, 2017 ▸). Each light source has different strong points, such as temporal resolution, the magnitude of pulse energy, and accessibility (Kraus *et al.*, 2018 ▸). Even though the accessibility of XFELs is limited, they can generate high pulse energy with a few mJ from a natural self-amplified spontaneous emission (SASE) beam (Δ*E*/*E* ≃ 5 × 10^−3^) (*i.e.* pink beam) and a few tens of µJ for a monochromatic one with wide photon energy (Emma *et al.*, 2010 ▸; Ishikawa *et al.*, 2012 ▸; Kang *et al.*, 2017 ▸). Photoinduced reactions such as isomerization, spin crossover, and intramolecular charge transfer have been studied using the femto­second X-ray pulses from XFELs (Wernet, 2019 ▸; Zhang *et al.*, 2017 ▸; Cordones *et al.*, 2018 ▸).

Tender X-rays have an energy range of 2 to 7 keV between the hard and soft X-ray energy regions (Axnanda *et al.*, 2015 ▸). This region includes the *K*-edges of light elements that are important in biochemical systems (*e.g.* S, P, Cl) and the *L*- or *M*-edges of heavy elements that include information about their electronic states and the ligand environment around them (Bearden & Burr, 1967 ▸; Czapla-Masztafiak *et al.*, 2016 ▸; Ochmann *et al.*, 2018 ▸; Smolentsev *et al.*, 2020 ▸; Van Kuiken *et al.*, 2017 ▸; Rehanek *et al.*, 2018 ▸; Nowak *et al.*, 2020 ▸; Abraham *et al.*, 2019 ▸; Biasin *et al.*, 2021 ▸; Rovezzi *et al.*, 2020 ▸; Cordones *et al.*, 2018 ▸; Ochmann *et al.*, 2017 ▸; Hormes *et al.*, 2021 ▸; Liu *et al.*, 2021 ▸). It is anticipated that the tender X-ray region has potential applicability to the study of largely unrevealed systems in biology, materials science, and chemistry. Whereas soft X-rays require a vacuum system, tender XAS can be performed under He ambient and thus can be implemented more easily than soft X-ray experiments.

Transition metal complexes that can undergo photoinduced reactions are a good model system for coupled electronic and nuclear dynamics (Huse *et al.*, 2010 ▸; Van Kuiken *et al.*, 2016 ▸; Cho *et al.*, 2016 ▸; Hong *et al.*, 2015 ▸). Tris(bi­pyridine)­ruthenium(II) chloride ([Ru(bpy)_3_]^2+^) is a prototypical intramolecular electron-transfer complex, the excited-state behavior of which has been extensively studied with optical spectroscopic methods (Gawelda *et al.*, 2006 ▸; Cannizzo *et al.*, 2006 ▸; Sato *et al.*, 2012 ▸; Damrauer *et al.*, 1997 ▸; Müller & Brettel, 2012 ▸). Visible-light excitation of [Ru(bpy)_3_]^2+^ leads to the formation of the singlet metal-to-ligand charge transfer (^1^MLCT) state which undergoes ultrafast (<100 fs) intersystem crossing to a triplet ^3^MLCT state (Yeh *et al.*, 2000 ▸; Bhasikuttan *et al.*, 2002 ▸; Damrauer *et al.*, 1997 ▸). The long-lived ^3^MLCT state decays radiatively to the ground state with 600 ns lifetime in water at room temperature (Creutz *et al.*, 1980 ▸). In the present study, we performed a femtosecond XAS measurement of aqueous [Ru(bpy)_3_]^2+^ pumped by visible laser pulses at the Pohang Accelerator Laboratory XFEL (PAL-XFEL). The present work expects to capture the ^3^MLCT state which can be unambiguously probed with XAS measurement at the Ru *L*_3_-edge. The newly developed experimental apparatus was set up for the measurements and the feasibility of femtosecond XAS in the tender X-ray range was evaluated. Our results indicate its capability for direct investigation of the femtosecond photoinduced dynamics of various systems.

## 2. Instrumentation

### 2.1. Hard X-ray beamline of PAL-XFEL

PAL-XFEL shows excellent performances in the beam position stability, energy jitter, time jitter, and coherence (Kang *et al.*, 2017 ▸; Yun *et al.*, 2019 ▸). The hard X-ray beamline at PAL-XFEL employs fixed-gap undulators so that the XFEL energy is tuned by changing the electron beam energy. Applying this operation mode enables a wide X-ray range from 2.2 to 20 keV. In particular, the tender X-ray range, which has attracted keen interest for chemistry and life science research using X-ray spectroscopy techniques, is available at the hard X-ray beamline of PAL-XFEL (Northrup *et al.*, 2016 ▸). Parameters of X-ray pulse are summarized in Table 1 ▸.

**Figure 1:**
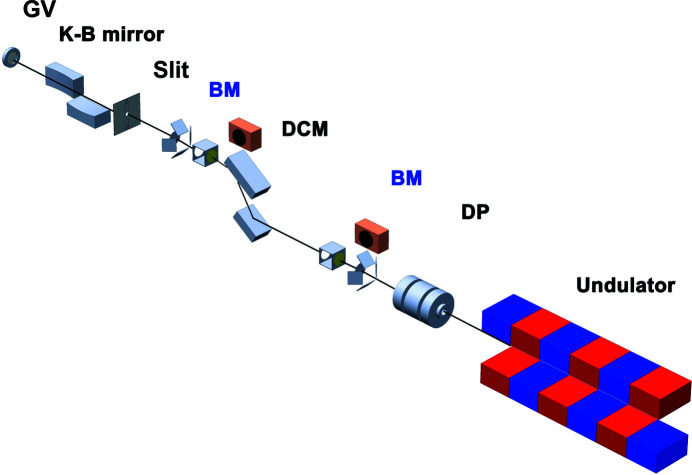
The layout of the hard X-ray beamline at the Pohang Accelerator Laboratory X-ray Free-Electron Laser (PAL-XFEL): differential pumping (DP), the beam monitor including the quadruple beam position monitor (BM), double-crystal monochromator (DCM), slit, a pair of Kirkpatrick–Baez (K-B) mirrors, and a gate valve (GV).

Fig. 1 ▸ shows the key components of the hard X-ray beamline. Optics and diagnostics are composed to deliver X-ray pulses to the experimental hutch while minimizing the disturbance of the wavefront and the loss of X-ray flux. An Si (111) double-crystal monochromator (DCM) is located at the optical hutch to provide higher energy resolution (Δ*E*/*E* ≃ 1 × 10^−4^) than a natural self-amplified spontaneous emission (SASE) beam (Δ*E*/*E* ≃ 5 × 10^−3^). Also, off-set mirrors are equipped for higher X-ray flux with a broad bandwidth, Δ*E*/*E* ≃ 5 × 10^−3^. Two options, DCM or off-set mirrors, are chosen by the preference of X-ray pulse properties, X-ray flux or narrow bandwidth. A differential pumping chamber with 6 mm apertures on both sides was adopted to separate the upstream hutch and the optical hutch without any X-ray transparent windows. Beam-position monitors (BPMs) and quadruple beam-position monitors (QBPMs; UHV QBPM DN100CF6", FMB Oxford) are located between each X-ray optic that reflects X-rays. The BPMs consist of a Y-Al garnet (YAG) crystal and a vision camera (Manta G-046B, Allied Vision Technologies GmbH). The QBPMs, which non-destructively measure the incident X-ray flux and the beam position using a thin film with back-scattering geometry, consist of four photodiodes and X-ray transparent windows made of silicon nitride (Si_3_N_4_) membranes and diamond films; both components can bypass the X-ray trajectory. A Kirk­patrick–Baez (K-B) mirror was used to deliver a micrometer-sized focused beam at the second experimental hutch (Kim *et al.*, 2018 ▸). Detailed specification of this K-B mirror is shown in Table 2 ▸. A four-jaw slit was located upstream of the K-B mirror to avoid scattering from the edge of the mirror. For a flexible sample environment, a 72 µm-thick Be window was mounted to separate the sample chamber and the high-vacuum area.

**Figure 2:**
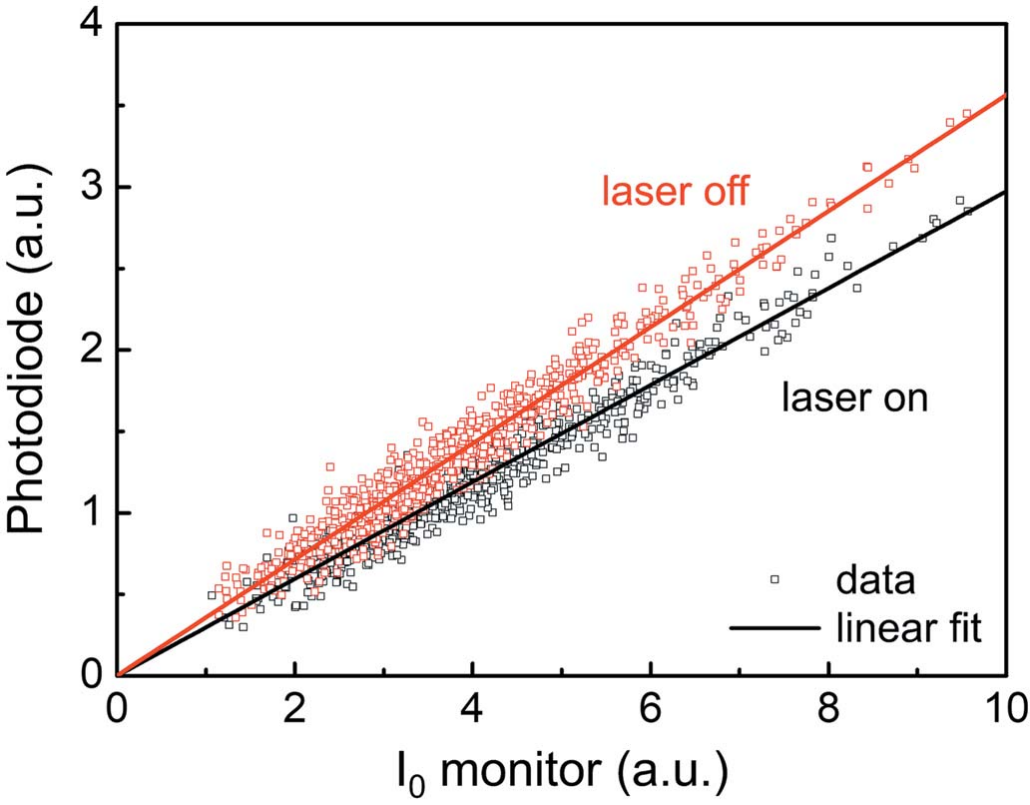
Correlation between the I_0_ monitor (QBPMs) and photodiode signals (X-ray fluorescence) obtained from an aqueous solution of tris­(bi­pyridine) ruthenium(II) chloride ([Ru(bpy)_3_]^2+^) at 2840.5 eV with (black squares) or without (red squares) the pump laser and their linear fits. QBPM: quadruple beam-position monitor.

Pulse energy fluctuation of the monochromated SASE FEL was normalized with the intensity of the QBPMs. A positive proportional relationship between the I_0_ monitor (QBPMs) and photodiode signals (X-ray fluorescence) was found for an aqueous solution of [Ru(bpy)_3_]^2+^ at 2840.5 eV with or without the pump laser (Fig. 2 ▸). The experimental conditions of this measurement will be described later in Section 3.2. A linear correlation between the I_0_ monitor and photodiode signal was obtained, and the slope of the linear fit was the normalized X-ray fluorescence. The slope with a laser was lower than that without because the transient peak of [Ru(bpy)_3_]^2+^ at 2840.5 eV was negative. Hence, the quality of the X-ray absorption near-edge structure (XANES) spectrum was improved by applying the normalization technique with the I_0_ intensity from QBPM.

### 2.2. X-ray absorption spectroscopy chamber

**Figure 3:**
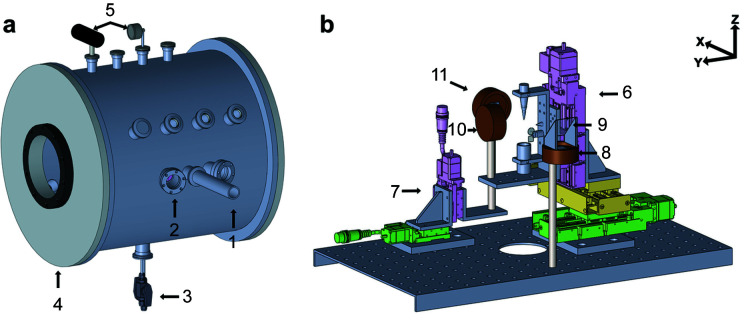
Schematics of (*a*) the X-ray absorption spectroscopy (XAS) chamber and (*b*) its inner components. The key components are marked with numbers: (1) the X-ray entrance port, (2) the laser entrance port, (3) the inlet and outlet for the liquid sample, (4) a hinged door, (5) two pressure meters (one digital and one analog), (6) the sample stage with *XYZ* translation stages [with a liquid nozzle, a pinhole, a Y-Al garnet (YAG) crystal, and a pinhole], (7) the detector stage with *YZ* translation stages, (8) a photodiode for recording incident flux, (9) 800 nm-thick Al foil as the X-ray scatterer for incident flux measurement, (10) an avalanche photodiode for measuring fluorescence from the sample, (11) a photodiode for measuring transmittance signals through the sample. The X-ray-free-electron laser (XFEL) beam direction follows the X-axis.

Fig. 3 ▸ shows a schematic view of the newly developed XAS chamber for time-resolved tender X-ray absorption experiments with the hard X-ray beamline at PAL-XFEL. Although it was designed to operate under ambient He, the vacuum environment, ∼low 10^−4^ torr, is also available. As shown in Fig. 3 ▸, the XAS chamber has three ports toward the inter­action region: one each at the incoming X-ray entrance, the pump laser entrance with an incidence angle of 15° to the XFEL beam, and for observing the interaction region using the long-distance microscope (UWZ500F; Union Optical Co. Ltd, Japan). Doors were attached on both sides of the chamber for easy access to mount samples and components inside the chamber; an extension can be achieved by removing one of the two doors and attaching a new chamber for other X-ray experiments such as X-ray emission spectroscopy. One viewport with a 176 mm diameter is on each door and another one with 130 mm diameter is on the rear side of the chamber for looking inside. Eighteen NW40 flanges and six NW25 flanges were installed to accommodate various configurations. A gas inlet port with a valve, a release port to prevent the chamber from overpressurization, and a port for a helium gas detector to monitor the concentration of the helium gas were connected. One Pirani and one analog pressure gauge were installed. To produce He ambient conditions, the chamber is pumped using a scroll pump first and then helium gas is injected into the chamber. Both pressure gauges operate alternatively for air and helium environments. Electric feedthrough flanges were attached to the rear side of the chamber to connect motorized stage cables and detector cables.

As shown in Fig. 3 ▸(*b*), the sample stage consists of three high-precision motorized stages for movement along the *X*, *Y*, and *Z* axes (Kohzu Precision, Japan). It has a liquid nozzle (Musashi Engineering, Japan) with a tip size of 150 µm, a reservoir for circulating the liquid sample, the YAG crystal for monitoring the XFEL beam position, and a pinhole to check the spatial overlap between the XFEL beam and the pump laser. An avalanche photodiode (APD; Excelitas technologies) was installed using a mounting rod on the plate attached to the *XYZ* stage to collect X-ray fluorescence signals from a sample. A photodiode (PD) (Hamamatsu) with a 10 mm^2^ active area for capturing transmission signals was mounted onto the *YZ* motorized stage for alignment with the XFEL pulses. Another PD was fixed to a mounting rod and employed to measure the incident X-ray flux (I_0_ intensity), which was used as a secondary I_0_ monitor (backup for QBPM). All the detectors were covered with two layers of 0.8 µm-thick Al foil to block unwanted signals from the pump laser scattered by the liquid sample. The transmission rate of X-rays at 2.84 keV through the 1.6 µm-thick Al foil was around 68% obtained from the Center for X-Ray Optics (CXRO). The current amplifiers (Femto DHPCA-100) were used for amplification of the PD and APD signals for I_0_ and the sample’s fluorescence signal. The bias voltage for each detector was supplied by using high-voltage suppliers (Keithley 248 High Voltage Supply). The PD signal was transported to the digitizer (NI PXIe-5160). Finally, 62% of the X-ray beam is illuminated to the sample, and 42% of the fluorescence signal is detected by the APD.

### 2.3. Pump laser system

A femtosecond laser system, as the pump laser, is available at the hard X-ray beamline, PAL-XFEL. This laser system consists of a Ti:sapphire oscillator (Vitara-T, Coherent, Inc.) and a regenerative amplifier with a single-pass amplifier (Legend Elite DUO HE, Coherent, Inc.). The fundamental wavelength of this system is 800 nm with 5 mJ maximum pulse energy at the interaction region. Recently the pulse duration has been set to 40 fs in FWHM using a pulse compressor. Additionally, a harmonic generator system and an optical parametric amplifier (OPA) are equipped to provide various wavelength ranges. The harmonic generator generates the second and the third harmonics, 400 nm and 266 nm. The tunable range of the OPA is from 240 nm to 2600 nm. This system provides a maximum 120 pulses s^−1^ (120 Hz). Table 3 ▸ shows a summary of parameters for the pump laser system.

### 2.4. Control software

**Figure 4:**
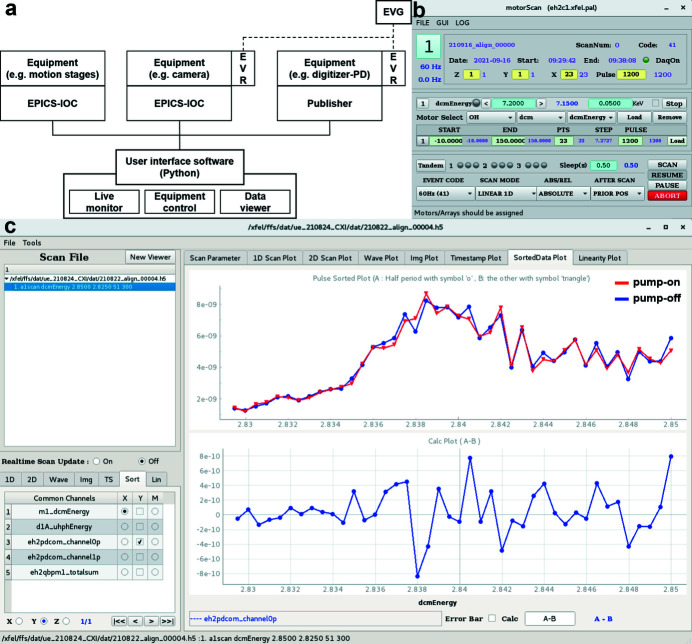
The DAQ and control software system at the PAL-XFEL. (*a*) A schematic diagram of the DAQ system at PAL-XFEL. Based on the event timing system, each X-ray pulse is named by a time-stamp and all operations are synchronized by trigger signals. Home-built control software transmit queues to operate single equipment or plural ones. (*b*) In-house developed main control panel. It executes queues for motion movement and data collection. (*c*) A panel of the data viewer. It displays collecting data in real time and provides a sorting function for pump–probe experiments. The upper graph shows pump-on and pump-off data together and the lower one displays the result of the arithmetic operation. Here, it shows the differences between two data sets.

PAL-XFEL generates 60 X-ray pulses in a second. Each X-ray pulse has different pulse energy due to the natural SASE fluctuations. It is essentially required to distinguish each X-ray pulse for data analysis. PAL-XFEL equipped an event-timing system, which consists of an event generator (EVG) timing master and event receiver (EVR) timing clients, to give a time-stamp (also called pulse ID) to each X-ray pulse. For example, a photodiode connected with a digitizer operates when they receive the trigger signal through the EVR. Based on this operation mode, all information is synchronously stored. X-ray detectors such as APD and PD in XAS experiments are connected with the digitizer and collect each data point with a time-stamp. Fig. 4 ▸(*a*) shows the overall concept of the PAL-XFEL data acquisition (DAQ) system briefly.

In-house control software was developed using Python [Fig. 4 ▸(*b*)]. Moving the motorized stages and data acquisition by the detectors were initiated by sending commands through the Experimental Physics and Industrial Control System (EPICS) Input/Output Controller (IOC) server or the publisher server. After movement of the motorized stages, feedback signals were transmitted to the control software through the EPICS IOC and then the measured data from detectors such as the QBPM and PDs were transferred from the publisher server to the control software via the Ethernet. This enabled recording of the intensity measured by the detectors at each motor position. The controller and interface unit were used to communicate between the EPICS IOC and motorized stages. Using this system at the PAL-XFEL enabled the remote operation of all equipment and acquisition of data using detectors while changing the position of the motorized stages or the incident X-ray energy. Also, the collected data points were displayed in real time through this software while executing scans.

A graphic user interface (GUI) based data viewer was prepared to check the data and carry out simple arithmetic operations [Fig. 4 ▸(*c*)]. For pump–probe experiments, it provided a sorting function to display pump-on and pump-off data separately. This function is useful to confirm the spatial overlap of two light sources and changes of the specimens by the pump laser. Further data analysis such as averaging and filtering was executed by Python-based codes. Advanced data analysis processes will be embodied by the GUI form.

## 3. Results and discussion

### 3.1. The energy-dependent path-length difference in the DCM

XAS measurements need a narrow energy bandwidth X-ray pulse from a DCM [a pair of Si (111) crystals with *ΔE*/*E* ≃ 1 × 10^−4^]. In the PAL-XFEL DCM case, the gap between the two crystals was adjusted based on the Bragg angle to maintain the same X-ray path before and after the DCM, which caused small changes in the X-ray path length in the DCM. This change had a minor effect on temporal resolution in a synchrotron experiment with a temporal resolution of around 70 ps. The path-length difference depends on the X-ray energy and the Bragg angle of the crystals inside the DCM: the larger the Bragg angle (lower the energy), the longer the path-length difference in unit energy. In the tender X-ray region, an energy difference of around 10 eV causes an X-ray arrival time difference into the sample of approximately 1 ps.

There are two ways of overcoming the path-length difference problem depending on X-ray energy: one is inline measurements of the X-ray and laser timing difference (the optical and X-ray temporal correlator at PAL-XFEL) and the other is to estimate the exact path-length difference inside the DCM and compensate for it in the laser path-length. The inline measurement using a timing diagnostic can monitor the X-ray arrival time of each X-ray pulse. The X-ray pulse should pass through the Si_3_N_4_ membrane. Two light sources pass through this membrane and the change of the transmittance of the pump laser is measured to identify the X-ray arrival time with femtosecond precision. Similar diagnostics were introduced and widely used in other XFEL facilities (Harmand *et al.*, 2013 ▸, 2014 ▸). Using a timing diagnostic is not efficient in the tender X-ray experiment. One main reason is that monochromatic X-ray flux (∼6 × 10^9^ photons pulse^−1^) is not enough to initialize the optical transmittance change. The other is that passing through Si_3_N_4_ (2 or 4 µm) reduces 40% or 64% of X-ray intensities. To collect temporal information, we need a thick membrane which is not optimum in real experiments in terms of incident X-ray flux.

**Figure 5:**
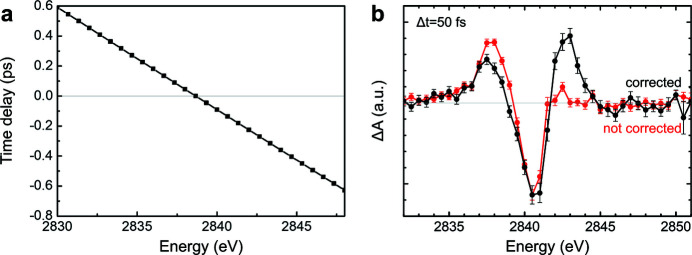
(*a*) Time delay difference as a function of incident X-ray energy at 2.84 keV. (*b*) Transient XAS spectrum of tris­(bi­pyridine) ruthenium(II) chloride ([Ru(bpy)_3_]^2+^) at a time delay of 50 fs before and after correcting for the X-ray arrival time through the double-crystal monochromator.

Instead of direct measuring, we applied the path-length compensation by a geometry analysis of the beam inside the DCM. We calculated the X-ray path length in the DCM and obtained an exit-time difference as a function of the incident X-ray energy. The timing diagnostic was used to validate our estimation by capturing real measurements in accumulation mode rather than shot-to-shot mode. The measurements show that our estimates were well matched with the real measurements as indicated in Fig. 5 ▸(*a*). At 2840 eV, a 10 eV difference caused a 700 fs arrival time difference.

We compared the transient XAS spectrum of [Ru(bpy)_3_]^2+^ at a time delay of 50 fs before and after correction, as shown in Fig. 5 ▸(*b*). The two transient curves are normalized by their negative peak values. At low energy (2837 eV), the positive peak of the uncorrected curve is higher than that of the corrected curve because the X-ray pulse arrived later than at the high energy, and so the time delay was shifted toward the positive side. At high energy (2842.5 eV), the second positive peak disappeared in the uncorrected curve because the X-ray pulse arrived earlier than at low energy, which moved the time delay toward the negative side.

### 3.2. Transient X-ray absorption spectrum of [Ru(bpy)_3_]^2+^ in water

The XAS experiment was conducted at the Nano-crystallography and Coherent Imaging (NCI) endstation of the PAL-XFEL. The incident photon energy was optimized at 2.84 keV, which corresponds to the vicinity of the Ru *L*_3_-edge, by tuning the electron beam energy. A DCM was used to achieve high energy resolution. A beam of ∼20 µm focused in both directions using a pair of K-B mirrors was delivered to the sample chamber. A slit located upstream of the K-B mirrors was opened to ∼1 mm × 1 mm to confirm that the beam position was incident to the K-B mirrors. The *L*_3_-edge of Ru was defined from the fluorescence signal generated from high-purity Ru foil with a thickness of 5 µm [extended X-ray absorption fine structure (EXAFS) standard foil; Exafs Materials]. Since the photon energy range for the energy scan is wider than the natural bandwidth of the SASE beam (∼0.5% and the PAL-XFEL-adapted fixed-gap undulator), the electron beam energy control system was applied to keep the XFEL flux at the maximum over the whole X-ray energy scan range.

**Figure 6:**
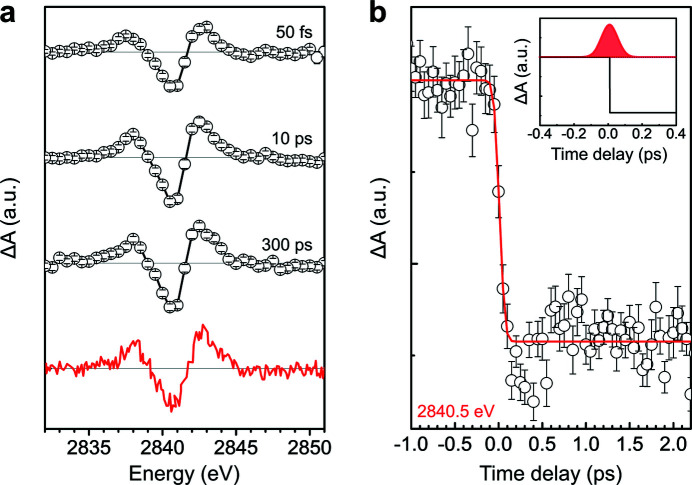
(*a*) Transient Ru *L*_3_-edge spectra of tris­(bi­pyridine) ruthenium(II) chloride ([Ru(bpy)_3_]^2+^) at time delays of 50 fs, 10 ps, and 300 ps (open circles and lines). The previously reported difference spectrum at 300 ps (red solid line) [reprinted with permission from Saes *et al.* (2003 ▸). Copyright (2003) by the American Physical Society]. (*b*) A kinetic trace of transient X-ray absorption spectroscopy (XAS) at the negative transient peak (2840.5 eV). The inset shows the Gaussian instrument response function and the step function resulting in the fitted curve.

Femtosecond TR-XAS measurements of a 50 m*M* aqueous Ru(bpy)_3_Cl_2_·6H_2_O (Sigma-Aldrich) solution were performed while controlling the photon energy of the X-ray pulse. The sample solution was circulated in a continuously flowing 150 µm-thick jet to avoid the accumulated damage from the X-ray and laser pulses. The sample was excited by using a 400 nm optical laser pulse of 100 fs duration (FWHM) at a repetition rate of 30 Hz produced by the second harmonic generation (SHG) of the 800 nm fundamental wavelength of the Ti:sapphire laser. The laser pulse was focused to 100 µm × 100 µm with 24 µJ at the sample position. The sample chamber was filled with helium, which was maintained under a continuous flow during the data acquisition, to minimize the loss of X-ray flux in the tender X-ray region. Transient X-ray absorption spectra of the Ru *L*_3_-edge were collected under total fluorescence yield (TFY) mode using an APD. Fig. 6 ▸(*a*) shows a comparison of the transient XAS spectrum of [Ru(bpy)_3_]^2+^ at time delays of 50 fs, 10 ps, and 300 ps with good signal-to-noise ratio and a previously reported difference spectrum at 300 ps. The shape of the transient spectra did not change with the time delay, which clearly indicates that the subpicosecond transient spectrum agrees with the picosecond pump–probe spectrum within the signal-to-noise ratio of the measurements. The difference spectrum measured at 50 fs delay indicates that the excited state with the new electronic configuration is formed within the instrument response function in this measurement. The difference spectrum for a 300 ps time delay exactly matches the previously reported spectrum corresponding to the formation of the metal-to-ligand charge transfer (MLCT) excited state [Fig. 6 ▸(*a*) red solid line] (Saes *et al.*, 2003 ▸).

The time evolution of the transient negative peak at 2840.5 eV is shown in Fig. 6 ▸(*b*). The difference signal increased sharply upon irradiation of the optical pulse. To calculate the temporal resolution for this experiment, the rise in transient signal was fitted with an error function which is a convolution of a Gaussian instrument response function and a step function; the estimated Gaussian instrument response function achieved a full width at half-maximum (FWHM) of around 140 fs. The temporal resolution is limited by the duration of the optical and X-ray pulses, timing jitter between optical pump and X-ray probe pulses, and the thickness of the liquid jet leading to the group velocity mismatch between optical and X-ray pulses. Considering the derived temporal resolution and results, this recently developed XAS chamber and setup at PAL-XFEL will facilitate femtosecond TR-XAS measurements in the tender X-ray region that will offer new insights into ultrafast photoinduced reactions.

## 4. Conclusion

We built an XAS chamber to facilitate the understanding of ultrafast photoinduced reactions of molecules at sub-ps temporal resolutions. This chamber was designed to be widely adaptable at two experimental hutches of the hard X-ray beamline at PAL-XFEL. TR-XAS in the tender X-ray region was performed with the newly developed experimental apparatus to provide a mechanistic picture of photoinduced electron transfer in [Ru(bpy)_3_]^2+^. These XAS experiments successfully demonstrated the feasibility of the newly developed experimental apparatus and setup. Furthermore, the application of femtosecond XANES will provide new insights into understanding ultrafast electron dynamics.

## Figures and Tables

**Table 1:** Summary of the key parameters of the photon beam

Photon energy range	2.2–20 keV
Bandwidth Δ*E*/*E* (SASE XFEL)	∼5 × 10^−3^ (natural bandwidth)
	∼1 × 10^−4^ [Si(111) double-crystal monochromator]
Maximum repetition rate	60 Hz
X-ray pulse duration	∼50 fs in FWHM
Photons per pulse at 10 keV (at the interaction region)	∼3 × 10^11^ (natural bandwidth)
	∼6 × 10^9^ [Si (111) double-crystal monochromator]
Photon beam size at 10 keV	∼300 µm × 300 µm in FWHM

**Table 2:** Summary of the key parameters for the focusing optics – the K-B mirror

Surface profile	Elliptical cylinder
Substrate material	Quartz
Surface coating	Bare for lower than 10 keV
	Rhodium for higher than 10 keV
Mirror size	600 mm × 50 mm × 50 mm (L × W × H)
Effective mirror area	588 mm × 22 mm (L × W) for vertical focusing mirror
	581 mm × 21 mm (L × W) for horizontal focusing mirror
Grazing incidence angle	2.6 mrad
Focal length	5.955 m for vertical focusing mirror
	5.365 m for horizontal focusing mirror
Spatial acceptance	1.53 mm for vertical focusing mirror
	1.51 mm for horizontal focusing mirror

**Table 3:** Summary of key parameters of the pump laser system – the femtosecond optical laser

Fundamental wavelength	800 nm
Maximum repetition rate	120 Hz
Pulse duration	40 fs in FWHM†
Maximum pulse energy at the sample position	3.0 mJ at 800 nm
	0.5 mJ at 400 nm
	0.3 mJ at 266 nm
Optical parametric amplifier	240–2600 nm

† 100 fs (FWHM) was used before upgrade in 2020.

## References

[bb1] Abraham, B., Nowak, S., Weninger, C., Armenta, R., Defever, J., Day, D., Carini, G., Nakahara, K., Gallo, A., Nelson, S., Nordlund, D., Kroll, T., Hunter, M. S., van Driel, T., Zhu, D., Weng, T.-C., Alonso-Mori, R. & Sokaras, D. (2019). *J. Synchrotron Rad.* **26**, 629–634. 10.1107/S1600577519002431 PMC6510194 31074425

[bb2] Attar, A. R., Bhattacherjee, A., Pemmaraju, C., Schnorr, K., Closser, K. D., Prendergast, D. & Leone, S. R. (2017). *Science*, **356**, 54–59. 10.1126/science.aaj2198 28386006

[bb3] Axnanda, S., Crumlin, E. J., Mao, B., Rani, S., Chang, R., Karlsson, P. G., Edwards, M. O., Lundqvist, M., Moberg, R., Ross, P., Hussain, Z. & Liu, Z. (2015). *Sci. Rep.* **5**, 9788. 10.1038/srep09788 PMC4650780 25950241

[bb4] Bearden, J. A. & Burr, A. (1967). *Rev. Mod. Phys.* **39**, 125–142.

[bb5] Bhasikuttan, A. C., Suzuki, M., Nakashima, S. & Okada, T. (2002). *J. Am. Chem. Soc.* **124**, 8398–8405. 10.1021/ja026135h 12105921

[bb6] Biasin, E., Nascimento, D. R., Poulter, B. I., Abraham, B., Kunnus, K., Garcia-Esparza, A. T., Nowak, S. H., Kroll, T., Schoenlein, R. W., Alonso-Mori, R., Khalil, M., Govind, N. & Sokaras, D. (2021). *Chem. Sci.* **12**, 3713–3725. 10.1039/d0sc06227h PMC8179428 34163645

[bb7] Bressler, C. & Chergui, M. (2004). *Chem. Rev.* **104**, 1781–1812. 10.1021/cr0206667 15080712

[bb9] Cammarata, M., Bertoni, R., Lorenc, M., Cailleau, H., Di Matteo, S., Mauriac, C., Matar, S. F., Lemke, H., Chollet, M., Ravy, S., Laulhé, C., Létard, J. F. & Collet, E. (2014). *Phys. Rev. Lett.* **113**, 227402. 10.1103/PhysRevLett.113.227402 25494090

[bb10] Cannizzo, A., van Mourik, F., Gawelda, W., Zgrablic, G., Bressler, C. & Chergui, M. (2006). *Angew. Chem.* **118**, 3246–3248. 10.1002/anie.200600125 16586519

[bb11] Chen, L. X. (2005). *Annu. Rev. Phys. Chem.* **56**, 221–254. 10.1146/annurev.physchem.56.092503.141310 15796701

[bb12] Chen, L. X., Zhang, X. & Shelby, M. L. (2014). *Chem. Sci.* **5**, 4136–4152.

[bb13] Cho, H., Hong, K., Strader, M. L., Lee, J. H., Schoenlein, R. W., Huse, N. & Kim, T. K. (2016). *Inorg. Chem.* **55**, 5895–5903. 10.1021/acs.inorgchem.6b00208 27248860

[bb14] Cordones, A. A., Lee, J. H., Hong, K., Cho, H., Garg, K., Boggio-Pasqua, M., Rack, J. J., Huse, N., Schoenlein, R. W. & Kim, T. K. (2018). *Nat. Commun.* **9**, 1989. 10.1038/s41467-018-04351-0 PMC5959936 29777157

[bb15] Creutz, C., Chou, M., Netzel, T. L., Okumura, M. & Sutin, N. (1980). *J. Am. Chem. Soc.* **102**, 1309–1319.

[bb16] Czapla-Masztafiak, J., Szlachetko, J., Milne, C. J., Lipiec, E., Sá, J., Penfold, T. J., Huthwelker, T., Borca, C., Abela, R. & Kwiatek, W. M. (2016). *Biophys. J.* **110**, 1304–1311. 10.1016/j.bpj.2016.01.031 PMC4816689 27028640

[bb17] Damrauer, N. H., Cerullo, G., Yeh, A., Boussie, T. R., Shank, C. V. & McCusker, J. K. (1997). *Science*, **275**, 54–57. 10.1126/science.275.5296.54 8974388

[bb18] De Groot, F. (2001). *Chem. Rev.* **101**, 1779–1808. 10.1021/cr9900681 11709999

[bb19] Emma, P., Akre, R., Arthur, J., Bionta, R., Bostedt, C., Bozek, J., Brachmann, A., Bucksbaum, P., Coffee, R., Decker, F.-J., Ding, Y., Dowell, D., Edstrom, S., Fisher, A., Frisch, J., Gilevich, S., Hastings, J., Hays, G., Hering, P., Huang, Z., Iverson, R., Loos, H., Messerschmidt, M., Miahnahri, A., Moeller, S., Nuhn, H., Pile, G., Ratner, D., Rzepiela, J., Schultz, D., Smith, T., Stefan, P., Tompkins, H., Turner, J., Welch, J., White, W., Wu, J., Yocky, G. & Galayda, J. (2010). *Nat. Photon.* **4**, 641–647.

[bb20] Gawelda, W., Johnson, M., de Groot, F. M., Abela, R., Bressler, C. & Chergui, M. (2006). *J. Am. Chem. Soc.* **128**, 5001–5009. 10.1021/ja054932k 16608334

[bb21] Harmand, M., Coffee, R., Bionta, M. R., Chollet, M., French, D., Zhu, D., Fritz, D., Lemke, H., Medvedev, N., Ziaja, B., Toleikis, S. & Cammarata, M. (2013). *Nat. Photon.* **7**, 215–218.

[bb22] Hartmann, N., Helml, W., Galler, A., Bionta, M., Grünert, J., Molodtsov, S., Ferguson, K., Schorb, S., Swiggers, M., Carron, S., Bostedt, C., Castagna, J., Bozek, J., Glownia, J. M., Kane, D. J., Fry, A. R., White, W. E., Hauri, C. P., Feurer, T. & Coffee, R. N. (2014). *Nat. Photon.* **8**, 706–709.

[bb23] Hong, K., Cho, H., Schoenlein, R. W., Kim, T. K. & Huse, N. (2015). *Acc. Chem. Res.* **48**, 2957–2966. 10.1021/acs.accounts.5b00154 26488127

[bb24] Hormes, J., Klysubun, W., Göttert, J., Lichtenberg, H., Maximenko, A., Morris, K., Nita, P., Prange, A., Szade, J., Wagner, L. & Zając, M. (2021). *Nucl. Instrum. Methods Phys. Res. B*, **489**, 76–81.

[bb25] Huse, N., Kim, T. K., Jamula, L., McCusker, J. K., de Groot, F. M. & Schoenlein, R. W. (2010). *J. Am. Chem. Soc.* **132**, 6809–6816. 10.1021/ja101381a 20426414

[bb26] Ishikawa, T., Aoyagi, H., Asaka, T., Asano, Y., Azumi, N., Bizen, T., Ego, H., Fukami, K., Fukui, T., Furukawa, Y., Goto, S., Hanaki, H., Hara, T., Hasegawa, T., Hatsui, T., Higashiya, A., Hirono, T., Hosoda, N., Ishii, M., Inagaki, T., Inubushi, Y., Itoga, T., Joti, Y., Kago, M., Kameshima, T., Kimura, H., Kirihara, Y., Kiyomichi, A., Kobayashi, T., Kondo, C., Kudo, T., Maesaka, H., Maréchal, X. M., Masuda, T., Matsubara, S., Matsumoto, T., Matsushita, T., Matsui, S., Nagasono, M., Nariyama, N., Ohashi, H., Ohata, T., Ohshima, T., Ono, S., Otake, Y., Saji, C., Sakurai, T., Sato, T., Sawada, K., Seike, T., Shirasawa, K., Sugimoto, T., Suzuki, S., Takahashi, S., Takebe, H., Takeshita, K., Tamasaku, K., Tanaka, H., Tanaka, R., Tanaka, T., Togashi, T., Togawa, K., Tokuhisa, A., Tomizawa, H., Tono, K., Wu, S., Yabashi, M., Yamaga, M., Yamashita, A., Yanagida, K., Zhang, C., Shintake, T., Kitamura, H. & Kumagai, N. (2012). *Nat. Photon.* **6**, 540–544.

[bb27] Kang, H.-S., Min, C.-K., Heo, H., Kim, C., Yang, H., Kim, G., Nam, I., Baek, S. Y., Choi, H.-J., Mun, G., Park, B. R., Suh, Y. J., Shin, D. C., Hu, J., Hong, J., Jung, S., Kim, S., Kim, K., Na, D., Park, S. S., Park, Y. J., Han, J., Jung, Y. G., Jeong, S. H., Lee, H. G., Lee, S., Lee, S., Lee, W., Oh, B., Suh, H. S., Parc, Y. W., Park, S., Kim, M. H., Jung, N., Kim, Y., Lee, M., Lee, B., Sung, C., Mok, I., Yang, J., Lee, C., Shin, H., Kim, J. H., Kim, Y., Lee, J. H., Park, S., Kim, J., Park, J., Eom, I., Rah, S., Kim, S., Nam, K. H., Park, J., Park, J., Kim, S., Kwon, S., Park, S. H., Kim, K. S., Hyun, H., Kim, S. N., Kim, S., Hwang, S., Kim, M. J., Lim, C., Yu, C., Kim, B., Kang, T., Kim, K., Kim, S., Lee, H., Lee, H., Park, K., Koo, T., Kim, D. & Ko, I. S. (2017). *Nat. Photon.* **11**, 708–713.

[bb28] Kapilashrami, M., Zhang, Y., Liu, Y.-S., Hagfeldt, A. & Guo, J. (2014). *Chem. Rev.* **114**, 9662–9707. 10.1021/cr5000893 25137023

[bb29] Kim, J., Kim, H.-Y., Park, J., Kim, S., Kim, S., Rah, S., Lim, J. & Nam, K. H. (2018). *J. Synchrotron Rad.* **25**, 289–292. 10.1107/S1600577517016186 PMC5741134 29271778

[bb30] Kraus, P. M., Zürch, M., Cushing, S. K., Neumark, D. M. & Leone, S. R. (2018). *Nat. Rev. Chem.* **2**, 82–94.

[bb31] Lemke, H. T., Bressler, C., Chen, L. X., Fritz, D. M., Gaffney, K. J., Galler, A., Gawelda, W., Haldrup, K., Hartsock, R. W., Ihee, H., Kim, J., Kim, K. H., Lee, J. H., Nielsen, M. M., Stickrath, A. B., Zhang, W., Zhu, D. & Cammarata, M. (2013). *J. Phys. Chem. A*, **117**, 735–740. 10.1021/jp312559h 23281652

[bb32] Liu, D.-G., Lee, M.-H., Lu, Y.-J., Lee, J.-F. & Chen, C.-L. (2021). *J. Synchrotron Rad.* **28**, 1202–1209. 10.1107/S1600577521003994 34212885

[bb33] Müller, P. & Brettel, K. (2012). *Photochem. Photobiol. Sci.* **11**, 632–636. 10.1039/c2pp05333k 22246402

[bb34] Northrup, P., Leri, A. & Tappero, R. (2016). *Protein Pept. Lett.* **23**, 300–308. 10.2174/0929866523666160107114505 26740327

[bb35] Nowak, S., Armenta, R., Schwartz, C., Gallo, A., Abraham, B., Garcia-Esparza, A., Biasin, E., Prado, A., Maciel, A., Zhang, D., Day, D., Christensen, S., Kroll, T., Alonso-Mori, R., Nordlund, D., Weng, T. C. & Sokaras, D. (2020). *Rev. Sci. Instrum.* **91**, 033101. 10.1063/1.5121853 32259983

[bb36] Ochmann, M., Hussain, A., von Ahnen, I., Cordones, A. A., Hong, K., Lee, J. H., Ma, R., Adamczyk, K., Kim, T. K., Schoenlein, R. W., Vendrell, O. & Huse, N. (2018). *J. Am. Chem. Soc.* **140**, 6554–6561. 10.1021/jacs.7b13455 29771112

[bb37] Ochmann, M., von Ahnen, I., Cordones, A. A., Hussain, A., Lee, J. H., Hong, K., Adamczyk, K., Vendrell, O., Kim, T. K., Schoenlein, R. W. & Huse, N. (2017). *J. Am. Chem. Soc.* **139**, 4797–4804. 10.1021/jacs.6b12992 28219243

[bb38] Rehanek, J., Milne, C. J., Szlachetko, J., Czapla-Masztafiak, J., Schneider, J., Huthwelker, T., Borca, C. N., Wetter, R., Patthey, L. & Juranić, P. (2018). *J. Synchrotron Rad.* **25**, 16–19. 10.1107/S1600577517012796 PMC5741116 29271745

[bb39] Rovezzi, M., Harris, A., Detlefs, B., Bohdan, T., Svyazhin, A., Santambrogio, A., Degler, D., Baran, R., Reynier, B., Noguera Crespo, P., Heyman, C., Van Der Kleij, H.-P., Van Vaerenbergh, P., Marion, P., Vitoux, H., Lapras, C., Verbeni, R., Kocsis, M. M., Manceau, A. & Glatzel, P. (2020). *J. Synchrotron Rad.* **27**, 813–826. 10.1107/S160057752000243X PMC7285681 32381786

[bb8] Saes, M., Bressler, C., Abela, R., Grolimund, D., Johnson, S., Heimann, P. & Chergui, M. (2003). *Phys. Rev. Lett.* **90**, 047403. 10.1103/PhysRevLett.90.047403 12570459

[bb40] Sato, T., Nozawa, S., Tomita, A., Hoshino, M., Koshihara, S., Fujii, H. & Adachi, S. (2012). *J. Phys. Chem. C*, **116**, 14232–14236.

[bb41] Schoenlein, R., Chattopadhyay, S., Chong, H., Glover, T., Heimann, P., Shank, C., Zholents, A. & Zolotorev, M. (2000). *Science*, **287**, 2237–2240. 10.1126/science.287.5461.2237 10731140

[bb42] Smolentsev, G., Milne, C. J., Guda, A., Haldrup, K., Szlachetko, J., Azzaroli, N., Cirelli, C., Knopp, G., Bohinc, R., Menzi, S., Pamfilidis, G., Gashi, D., Beck, M., Mozzanica, A., James, D., Bacellar, C., Mancini, G. F., Tereshchenko, A., Shapovalov, V., Kwiatek, W. M., Czapla-Masztafiak, J., Cannizzo, A., Gazzetto, M., Sander, M., Levantino, M., Kabanova, V., Rychagova, E., Ketkov, S., Olaru, M., Beckmann, J. & Vogt, M. (2020). *Nat. Commun.* **11**, 2131. 10.1038/s41467-020-15998-z PMC7195477 32358505

[bb43] Van Kuiken, B. E., Cho, H., Hong, K., Khalil, M., Schoenlein, R. W., Kim, T. K. & Huse, N. (2016). *J. Phys. Chem. Lett.* **7**, 465–470. 10.1021/acs.jpclett.5b02509 26727390

[bb44] Van Kuiken, B. E., Ross, M. R., Strader, M. L., Cordones, A. A., Cho, H., Lee, J. H., Schoenlein, R. W. & Khalil, M. (2017). *Struct. Dyn.* **4**, 044021. 10.1063/1.4983157 PMC5422206 28529962

[bb45] Wernet, P. (2019). *Philos. Trans. R. Soc. A.* **377**, 20170464. 10.1098/rsta.2017.0464 PMC6452048 30929622

[bb46] Yeh, A. T., Shank, C. V. & McCusker, J. K. (2000). *Science*, **289**, 935–938. 10.1126/science.289.5481.935 10937993

[bb47] Yun, K., Kim, S., Kim, D., Chung, M., Jo, W., Hwang, H., Nam, D., Kim, S., Kim, J., Park, S.-Y., Kim, K. S., Song, C., Lee, S. & Kim, H. (2019). *Sci. Rep.* **9**, 3300. 10.1038/s41598-019-39765-3 PMC6397240 30824784

[bb48] Zhang, W., Kjaer, K. S., Alonso-Mori, R., Bergmann, U., Chollet, M., Fredin, L. A., Hadt, R. G., Hartsock, R. W., Harlang, T., Kroll, T., Kubiček, K., Lemke, H. T., Liang, H. W., Liu, Y., Nielsen, M. M., Persson, P., Robinson, J. S., Solomon, E. I., Sun, Z., Sokaras, D., van Driel, T. B., Weng, T. C., Zhu, D., Wärnmark, K., Sundström, V. & Gaffney, K. J. (2017). *Chem. Sci.* **8**, 515–523. 10.1039/c6sc03070j PMC5341207 28451198

